# Relationship between self-perceptions of ageing and interoception among older people living with HIV: a latent profile analysis

**DOI:** 10.1186/s12889-026-27941-w

**Published:** 2026-05-28

**Authors:** Ting Yang, Chengde Su, Qiaoqiao Yao, Qianqian Zhu, Yali Xu, Mingdan Li, Zhiyan Bao, Huajun Wang, Qiuxiang Li, Ping Yang

**Affiliations:** 1 Department of Infectious Diseases, Affiliated Hospital of Zunyi Medical University, Zunyi, 563000 China; 2 Department of Nursing, Affiliated Hospital of Zunyi Medical University, Zunyi, 563000 China; 3 School of Nursing, Zunyi Medical University, Zunyi, 563000 China

**Keywords:** Older adults, PLWH, Self-perceptions of ageing, Interoception, Health ecological model, Latent profile analysis

## Abstract

**Background:**

Older people living with HIV (PLWH) commonly experience accelerated ageing; however, their subjective experiences of ageing vary substantially among individuals. Self-perceptions of ageing (SPA) is an important psychological factor influencing physical and mental health in older adults, while interoception reflects the ability to perceive and interpret internal bodily signals. To date, the latent heterogeneity of SPA and its relationship with interoception among older PLWH remain unclear. Therefore, this study aimed to identify latent profiles of SPA, examine associated factors, and explore the association between SPA and interoception among older PLWH.

**Methods:**

A cross-sectional study was conducted from July to December 2025 among 269 older PLWH recruited by convenience sampling from a tertiary hospital in Zunyi. Data were collected using a general information questionnaire, the Brief Ageing Perceptions Questionnaire (B-APQ), the Multidimensional Assessment of Interoceptive Awareness Version 2 (MAIA-2), the FRAIL scale, and the Social Support Rating Scale (SSRS). Latent Profile Analysis was used to identify latent SPA profiles, and multinomial logistic regression was performed to examine factors associated with profile membership. In addition, quantile regression, Restricted Cubic Spline (RCS) regression, and Bayesian factor robustness tests were conducted to evaluate the relationship between SPA and interoception.

**Results:**

Three latent SPA profiles were identified: low SPA (*n* = 43, 15.985%), moderate SPA (*n* = 60, 22.305%), and high SPA (*n* = 166, 61.710%). Educational level, employment status, personal monthly income, alcohol history, and frailty were significant factors associated with SPA profile membership. Compared with the low SPA group, participants in the high SPA group had significantly lower total interoception scores and lower scores across all interoceptive dimensions (*P* < 0.001). RCS regression showed a significant non-linear negative association between SPA and interoception (*P* = 0.023), with a potential threshold at a B-APQ score of 25.55. When SPA scores reached or exceeded this threshold, interoceptive ability declined more markedly. Bayesian factor analysis further supported the robustness of the differences in interoception across SPA profiles.

**Conclusions:**

SPA among older PLWH shows substantial heterogeneity and is jointly influenced by individual characteristics, behavioral factors, and socioeconomic conditions. SPA was negatively and non-linearly associated with interoception, suggesting that higher levels of negative ageing perceptions may be related to poorer awareness of internal bodily signals. Healthcare providers should implement stratified health management and individualized interventions based on SPA subtypes to improve ageing perceptions and promote healthy ageing among older PLWH.

**Clinical trial number:**

Not applicable.

## Background

With the widespread use of antiretroviral therapy (ART), HIV/AIDS has evolved from a fatal disease into a chronic condition that can be effectively treated and managed over the long term, resulting in substantially increased life expectancy among people living with HIV (PLWH) [1, 2]. According to the 2025 report from the Joint United Nations Programme (UNAIDS), approximately 40.8 million people worldwide were living with HIV [3]. Among them, individuals aged 50 years and older are internationally defined as older PLWH, and this population has reached 7.9 million, accounting for 21% of all PLWH globally [4, 5]. In China, population ageing among PLWH is even more pronounced, with older PLWH accounting for 34% of all surviving PLWH nationwide [6, 7]. In this context, promoting positive perceptions of ageing among older PLWH has important public health implications for achieving healthy ageing.

Self-perceptions of ageing (SPA) refers to individuals’ subjective perceptions and emotional responses to the biological, psychological, and social challenges associated with ageing [8]. According to the Stereotype Embodiment Theory (SET) [9], individuals gradually internalize age-related stereotypes throughout the life course, and these internalized beliefs are transformed into attitudes and expectations regarding their own ageing process in later life. Once individuals identify themselves as “older adults,” these positive or negative internalized age stereotypes may shape their SPA and subsequently influence mental health [10]. Previous studies have shown that positive SPA contributes to successful ageing and is associated with improved quality of life, better physical and mental health, and longer survival [11–13]. In contrast, negative SPA has been linked to physical functional decline, poorer psychological health, and increased negative emotional experiences [14]. Among older adults with chronic diseases, negative perceptions of ageing may further contribute to pessimistic attitudes and cumulative health risks [15, 16].

Among older PLWH, SPA may present more complex characteristics. Due to long-term HIV infection and persistent chronic inflammation, PLWH often exhibit accelerated immunosenescence, with biological ageing frequently exceeding chronological age and earlier onset of geriatric syndromes such as frailty and multimorbidity [17]. In addition, older PLWH may simultaneously experience HIV-related stigma and ageism, resulting in a dual stigma burden [18]. This dual marginalization may lead individuals to reduce social participation and interpersonal interactions, thereby exacerbating social isolation and weakening positive ageing experiences [19, 20]. Therefore, exploring the characteristics and influencing factors of SPA among older PLWH is essential for promoting healthy ageing in this population.

Interoception refers to the process by which individuals perceive, interpret, integrate, and regulate internal bodily signals, such as heartbeat, respiration, and gastrointestinal sensations [21]. It plays a key role in emotional regulation, body representation, and self-awareness [22, 23]. Existing evidence suggests a potential association between SPA and interoception. The anterior insula and anterior cingulate cortex, which are core brain regions involved in interoceptive processing, also contribute to emotional regulation and self-related cognition [24]. Negative perceptions of ageing may impair attention to and interpretation of internal bodily signals through pathways related to emotional dysregulation, thereby reducing interoceptive ability [25]. Previous studies have indicated that older adults with anxiety about ageing may exhibit attentional bias toward bodily signals or blunted bodily perception, whereas positive attitudes toward ageing are associated with greater bodily awareness [26, 27]. Furthermore, even under viral suppression, HIV infection is characterized by persistent chronic inflammation, which may accelerate brain ageing and impair the functional integrity of interoception-related brain regions such as the anterior insula [28]. Therefore, under the context of HIV-related accelerated ageing, the relationship between SPA and interoception may be more complex than that observed in the general older population. However, this relationship has not yet been specifically examined in older PLWH.

Most existing studies on SPA among PLWH have adopted variable-centered approaches, typically relying on total scale scores to assess ageing perceptions, which may overlook underlying heterogeneity across individuals. Latent Profile Analysis (LPA) is a person-centered latent variable approach that identifies distinct subgroups based on patterns across multiple continuous indicators [29]. In recent years, LPA has been increasingly used to identify different SPA subtypes among older adults. For example, one cross-sectional study identified three SPA profiles among community-dwelling older adults in China: “low ageing awareness with high positive control,” “low positive consequences and control,” and “high ageing awareness with negative control” [30]. In addition, Liu et al. identified two SPA developmental trajectories (“high-level group” and “low-level group”) using data from the Chinese Longitudinal Healthy Longevity Survey [31]. Although SPA has been investigated in general older populations, evidence regarding SPA heterogeneity among older PLWH remains limited. Given that SPA is closely associated with physical functioning, mental health, and longevity [32], identifying distinct SPA subtypes and their associated risk factors among older PLWH is important for early screening and targeted interventions.

Previous studies have shown that SPA is influenced by multiple factors, including individual characteristics and external environmental conditions. For instance, a study conducted among Chinese older adults found that sex, age, educational level, type of medical insurance, and frequency of children’s visits were significantly associated with SPA [33]. In addition, marital status, place of residence, social networks, outdoor activity time, chronic diseases, and psychological conditions have also been reported as relevant factors [34, 35]. However, whether these factors similarly affect SPA among older PLWH remains unclear. Although previous studies have identified factors at different levels, few have integrated these influences into a coherent analytical framework to explain how they jointly shape SPA [36].

To address these gaps, this study adopted the Health Ecological Model (HEM) as the theoretical framework to examine factors associated with SPA among older PLWH. The HEM conceptualizes health as the result of interactions between individual and environmental factors across five levels: personal traits, behavioral characteristics, interpersonal networks, living and working conditions, and policy environment [37]. This framework emphasizes the complexity and multidimensionality of health determinants and has been effectively applied in studies involving PLWH [38, 39]. Guided by the HEM, this study integrated individual characteristics and external environmental factors into a unified empirical framework to systematically analyze the determinants of SPA among older PLWH, thereby providing evidence for developing targeted psychosocial interventions and promoting healthy ageing in this population.

This study had two main objectives: (1) to identify latent SPA profiles among older PLWH using LPA and to examine associated factors based on the HEM; and (2) to further explore the relationship between SPA and interoception under the guidance of SET. These findings may provide a theoretical basis and practical evidence for identifying high-risk individuals and developing individualized intervention strategies.

## Methods

### Study design and participants

This cross-sectional study was conducted from July to December 2025. Participants were recruited using convenience sampling from a tertiary hospital in Zunyi, Guizhou Province, China. The study population comprised older PLWH.

The inclusion criteria were as follows: (1) meeting the diagnostic criteria for HIV/AIDS as outlined in the Chinese Guidelines for the Diagnosis and Treatment of HIV/AIDS (2024 edition) [40]; (2) aged 50 years or older [41]; (3) being aware of their HIV diagnosis; and (4) being able to communicate normally and willing to participate in the study. The exclusion criteria were: (1) having severe cognitive impairment (e.g., dementia or Alzheimer’s disease) or severe psychiatric disorders (including Schizophrenia, Post-traumatic stress disorder, and Bipolar disorder) diagnosed by a clinical psychiatrist; (2) having other severe physical illnesses; and (3) having sensory impairments (e.g., visual or hearing impairment) that prevented the provision of valid information.

### Sample size calculation

**Fig. 1:**
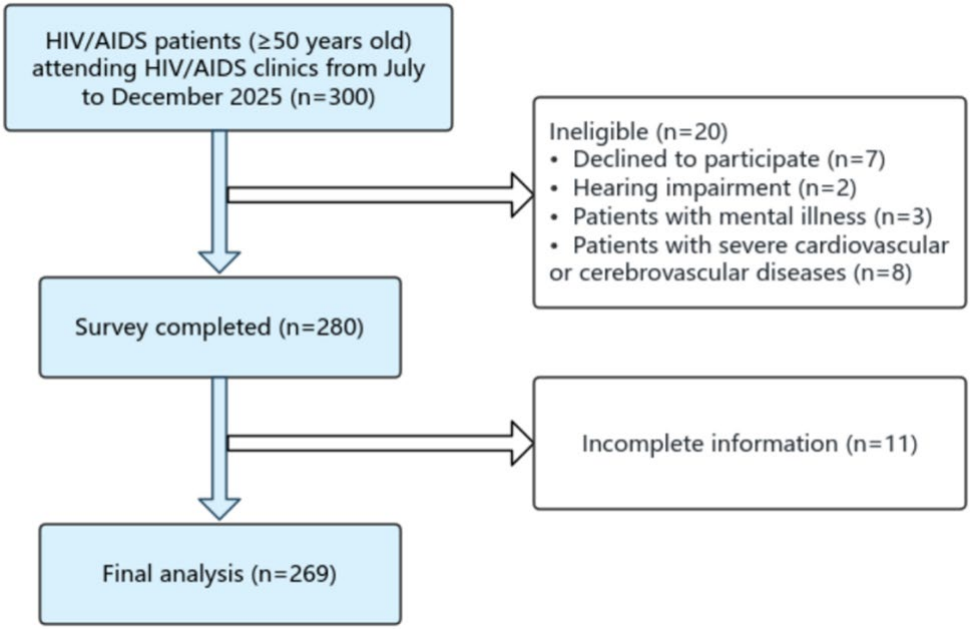
Participant flow diagram

The sample size was estimated using Kendall’s rule of thumb, which recommends a sample size of 5–10 times the number of variables included in the analysis [42]. A total of 38 variables were included in this study, comprising 21 general demographic variables and 17 scale dimensions (derived from four instruments). After accounting for a 20% potential invalid response rate, the required sample size was estimated to range from 238 to 475 participants. A total of 269 participants were ultimately included in the final analysis. The participant recruitment process is shown in Fig. 1.

This study was approved by the Ethics Committee of Affiliated Hospital of Zunyi Medical University (approval number: KLLY-2023-714). Written informed consent was obtained from all participants prior to data collection. The study was conducted in accordance with the principles of the Declaration of Helsinki.

### Instruments

#### General information questionnaire

**Fig. 2:**
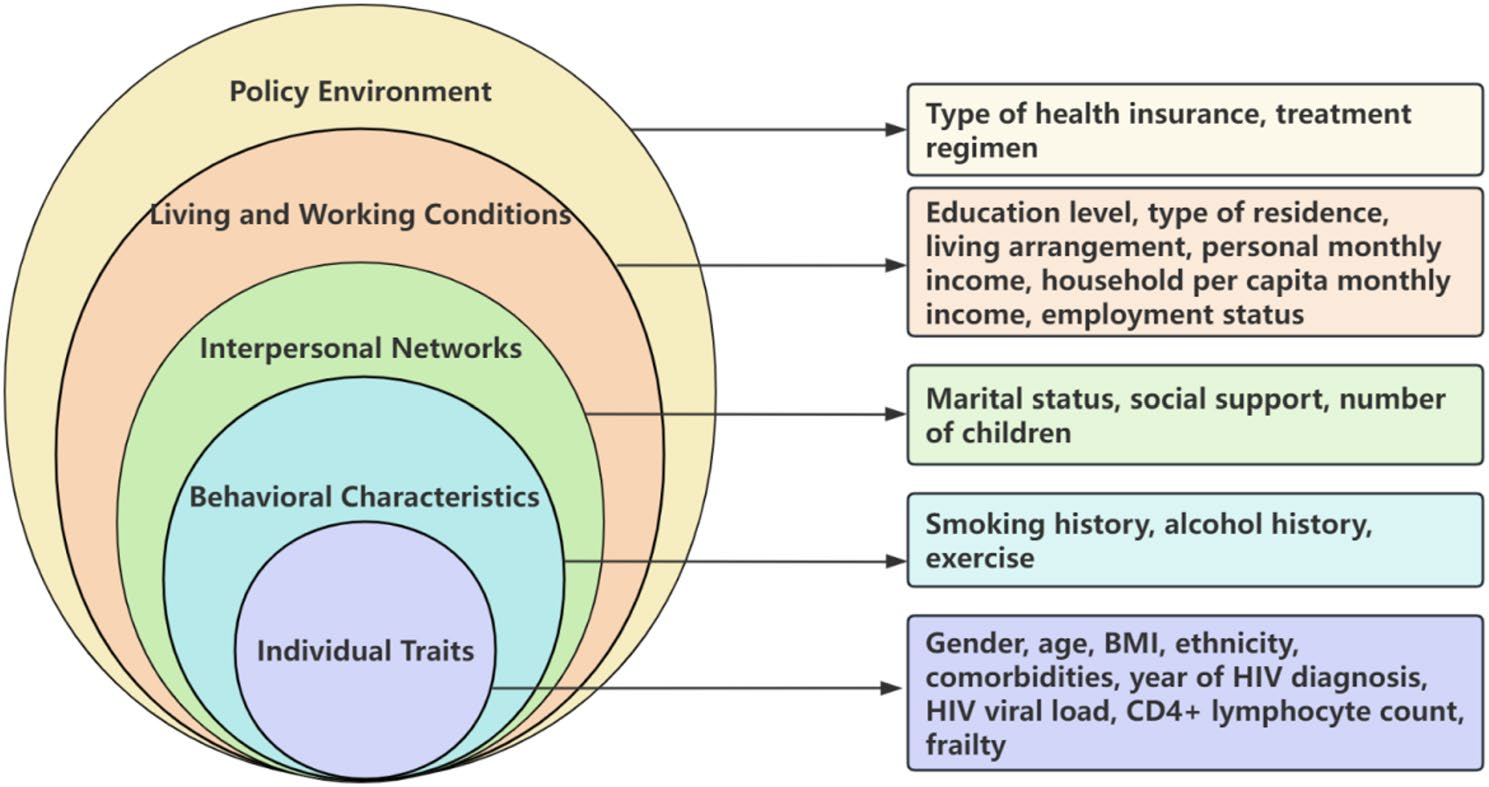
Relationship between independent variables and the HEM

Based on the HEM [37], this study examined factors associated with SPA among older PLWH. Independent variables were selected according to the five dimensions of the HEM, including the individual traits layer, behavioral characteristics layer, interpersonal networks layer, living and working conditions layer, and policy environment layer. The relationships between the independent variables and the HEM framework are presented in Fig. 2. Detailed definitions and coding of all independent variables are shown in Table 1. 

**Table 1:** Independent variables selected based on HEM and their assignments

Based on HEM Dimensions	Observation Variables	Assignment Method
Personal Traits	Gender	Male = 1, Female = 2
	Age	50–60 = 1, 61–70 = 2, ≥ 71 = 3
	BMI	< 18.5 = 1, 18.5–25.0 = 2, ≥ 25.0 = 3
	Ethnicity	Han = 1, Ethnic Minority = 2
	Complications	0 types = 0, 1 type = 1, 2 or more types = 2
	HIV Diagnosis Year	< 3 years = 1, 3–5 years = 2, > 5 years = 3
	HIV Viral Load	≤ 200 = 1, > 200 = 2
	CD4 + Lymphocyte Count	≤ 200 = 1, 201–500 = 2,≥500 = 3
	Frailty	Output original value
Behavioral Characteristics	Smoking History	No = 0, Yes = 1
	Drinking History	No = 0, Yes = 1
	Exercise Frequency (≥ 30 min/day)	Daily = 1, Often = 2, Occasionally = 3, Never = 4
Social Networks	Marital Status	Married = 1, Divorced = 2, Widowed = 3, Unmarried = 4
	Number of Children	≤ 2 = 1, > 2 = 2
	Social Support	Output original value
Living and Working Conditions	Education Level	Primary school = 1, Junior high school = 2, High school and above = 3
	Residence Type	Urban = 1, Urban-rural = 2, Rural = 3
	Living Arrangement	With spouse = 1, With children/parents = 2, Other = 3
	Personal Monthly Income	≤ 1000 = 1, 1001–3000 = 2, ≥ 3001 = 3
	Household Monthly Income per Capita	≤ 1000 = 1, 1001–3000 = 2, ≥ 3001 = 3
	Employment Status	Employed = 1, Retired = 2, Unemployed = 3
Policy Environment	Health Insurance Type	Urban Resident Insurance = 1, Employee Insurance = 2
	Treatment Plan	EFV/TDF/3TC = 1, Biktarvy = 2, Other = 3

EFV/TDF/3TC: Efavirenz + Tenofovir + Lamivudine

Several variables were defined as follows: (1) Smoking history was defined as current or previous smoking of at least one cigarette per day for a duration of 6 months or longer [43]; (2) alcohol history was defined as drinking at least twice per month, with each episode involving ≥ 20 g of pure ethanol (equivalent to approximately 500 mL of beer, 50 mL of spirits, or 150 mL of wine) [43]; (3) body mass index (BMI) was calculated as weight (kg) divided by height squared (m²) and categorized as underweight (< 18.5 kg/m²), normal weight (18.5–25.0 kg/m²), and overweight or above (≥ 25.0 kg/m²); (4) physical activity was classified as never (0 times/week), occasionally (1–2 times/week), frequently (3–6 times/week), and daily (≥ 7 times/week); and (5) comorbidities referred to the self-reported number of chronic diseases, including but not limited to Hypertension, Diabetes mellitus, Stroke, and Coronary heart disease.

#### Self-perceptions of ageing

SPA were assessed using the Brief Ageing Perceptions Questionnaire (B-APQ), which was developed by Sexton et al. in 2014 [44], and later translated into Chinese and validated for use among community-dwelling older adults by Hu Na [12]. The B-APQ consists of 17 items across five dimensions: timeline chronic (3 items), positive consequences (3 items), positive control (3 items), negative consequences and control (5 items), and emotional representations (3 items). Each item is rated on a 5-point Likert scale ranging from 1 (“strongly disagree”) to 5 (“strongly agree”). Items 4, 5, 6, 8, 9, and 10 are reverse scored. The total score ranges from 17 to 85, with higher scores indicating more negative self-perceptions of ageing.The Chinese version of the B-APQ has demonstrated good psychometric properties in older populations, with a Cronbach’s α of 0.914 [12], and has also been applied in studies involving older adults with chronic diseases [45]. In the present study, the Cronbach’s α coefficient for the B-APQ was 0.879, indicating good internal consistency.

#### Interoception

Interoception was assessed using the Multidimensional Assessment of Interoceptive Awareness, Version 2 (MAIA-2) in older PLWH. The MAIA-2 was developed by Wolf E. Mehling in 2018 as a revised version of the original Multidimensional Assessment of Interoceptive Awareness (MAIA) [46], and was subsequently translated into Chinese by Teng et al. [47]. The MAIA-2 comprises 37 items across eight dimensions: noticing, not-distracting, not-worrying, attention regulation, emotional awareness, self-regulation, body listening, and trusting. Items 5, 6, 8, 9, 10, 11, and 15 are reverse scored. Each item is rated on a 6-point Likert scale ranging from 0 (“never”) to 5 (“always”). The mean score of each dimension was calculated, and the sum of the mean scores across all eight dimensions was used as the total score. Higher scores indicate stronger awareness, interpretation, and integration of internal bodily signals.In the present study, the Cronbach’s α coefficient for the MAIA-2 was 0.987, indicating excellent reliability.

#### Frailty

Frailty was assessed using the FRAIL scale, which was recommended by the Chinese Expert Consensus on Frailty Prevention in Older Adults (2022) [48]. The scale includes five items: (1) In the past month, have you been unable to complete things you wanted to do due to physical exhaustion? (2) Can you climb one flight of stairs without assistance? (3) Can you walk 100 m continuously without assistance? (4) Have you been diagnosed with 5 or more of the following diseases? Hypertension, stroke, acute heart disease event, diabetes, congestive heart failure, chronic lung disease, arthritis, osteoporosis, angina, kidney disease, cancer, others; (5) Have you experienced a decreased appetite or significantly reduced food intake compared to before? Each item is scored dichotomously (yes = 1, no = 0), with a total score ranging from 0 to 5. A score of 0 indicates no frailty, 1–2 indicates pre-frailty, and ≥ 3 indicates frailty. The FRAIL scale has been widely used in studies involving PLWH [49, 50]. In the present study, the Cronbach’s α coefficient was 0.755, indicating acceptable internal consistency.

#### Social support

Social support was assessed using the Social Support Rating Scale (SSRS), developed by Xiao Shuiyuan in 1986 [51], to measure the level of social support among older PLWH. The SSRS consists of 10 items across three dimensions: subjective support (4 items), objective support (3 items), and support utilization (3 items). The total score is the sum of all item scores, ranging from 12 to 66. A score of 0–22 indicates low social support, 23–44 indicates moderate social support, and 45–66 indicates a satisfactory level of social support. The SSRS has demonstrated good reliability and validity, with Cronbach’s α coefficients ranging from 0.890 to 0.940. Its validity in Chinese populations of PLWH has been previously confirmed [38]. In the present study, the Cronbach’s α coefficient of the SSRS was 0.791, indicating good internal consistency.

### Data collection and quality control

To ensure data quality, two trained researchers were involved in the data collection process. First, one researcher individually explained the study’s objectives, significance, and procedures to each participant. After obtaining written informed consent, the second researcher guided participants to a quiet and private room for one-to-one interviews. Participants were then asked to complete the questionnaires independently, with neutral instructions provided and no leading or suggestive language used during the process. For participants who were illiterate or unable to complete the questionnaires independently, trained researchers read each item aloud in a private setting and recorded their responses verbatim.

A total of 300 questionnaires were distributed. After data collection, all questionnaires were carefully checked by investigators. Questionnaires were excluded if they had more than 20% missing items, showed obvious patterned responses, or contained logical inconsistencies. Finally, 269 valid questionnaires were included in the analysis, yielding an effective response rate of 89.7%. All participants took part voluntarily and did not receive any financial compensation.

### Statistical analysis

All data analyses were performed using SPSS 29.0, Mplus 8.3, JASP 0.95.4, and R 4.3.1.

Data analysis was conducted in four steps. First, due to the non-normal distribution of the data (*P* < 0.05), therefore, continuous variables were presented as medians and interquartile ranges [M (P25, P75)]. Group comparisons were performed using the Kruskal–Wallis H test. Categorical variables were summarized as frequencies and percentages and compared using the chi-square (χ²) test.

Second, LPA was conducted to identify SPA subgroups among PLWH. Models with increasing numbers of classes were fitted sequentially until no further significant improvement in model fit was observed. Model selection was based on multiple fit indices, including the Akaike Information Criterion (AIC), Bayesian Information Criterion (BIC), adjusted Bayesian Information Criterion (aBIC), entropy, Lo–Mendell–Rubin likelihood ratio test (LMR), and bootstrap likelihood ratio test (BLRT). (1) Lower AIC, BIC, and aBIC values indicate better model fit [52]; (2) entropy values range from 0 to 1, with values closer to 1 indicating higher classification accuracy [53, 54]; (3) significant LMR and BLRT results (*P* < 0.05) indicate that a k-class model fits significantly better than a k − 1-class model [55]; and (4) the final model selection also considered class size adequacy and clinical interpretability. To avoid potential classification bias, each class was required to include at least 10% of the total sample [56, 57].

Third, after the classification was completed, univariate analyses were conducted for categorical variables. Multicollinearity among independent variables was assessed using the variance inflation factor (VIF), with all VIF values < 10, indicating no significant multicollinearity. Subsequently, using the first latent class as the reference group, multinomial logistic regression was performed to identify factors associated with SPA class membership.

Finally, to examine the association between SPA classes and interoception, multivariate quantile regression was conducted. SPA class membership was used as the independent variable, with the first class as the reference group, and total MAIA-2 scores and subscale scores as dependent variables at the 50th percentile (median). In addition, Bayesian factor (BF10) analysis was used to assess the robustness of between-group differences, where BF10 > 10 indicates strong evidence for the alternative hypothesis, 4–10 indicates moderate evidence, and 1–3 indicates weak evidence [58]. Furthermore, restricted cubic spline (RCS) regression with knots at the 10th, 50th, and 90th percentiles was used to flexibly model the relationship between SPA and interoception. Regression coefficients (β) and 95% confidence intervals (95% CI) were calculated to estimate dose–response associations. A P-value < 0.05 for nonlinearity indicated a nonlinear relationship; otherwise, a linear relationship was assumed. A two-sided *P* < 0.05 was considered statistically significant for all analyses.

## Results

### Baseline characteristics of participants

A total of 269 older PLWH were included in this study. Of these, 102 (37.9%) were female and 167 (62.1%) were male. The age of participants ranged from 50 to 90 years.Regarding BMI, 65.8% (*n* = 177) of participants were within the normal range, followed by overweight or above (29.7%, *n* = 80) and underweight (4.5%, *n* = 12). Most participants were of Han ethnicity (94.1%, *n* = 253), lived in urban areas (48.0%, *n* = 129), were married (65.8%, *n* = 177), and lived with their spouse (42.4%, *n* = 114). Other baseline characteristics are presented in Table 2.

**Table 2:** Participant characteristics and univariate analysis (*n* = 269)

Variable	Total (*n* = 269)	Class1 (*n* = 43)	Class2 (*n* = 60)	Class3 (*n* = 166)	χ²/H value	*P*-value
Gender					1.298^a^	0.523
Male	167 (62.1)	30 (69.8)	36 (60.0)	101 (60.8)		
Female	102 (37.9)	13 (30.2)	24 (40.0)	65 (39.2)		
Age					21.948^a^	< 0.001
50–60	131 (48.7)	30 (69.8)	38 (63.3)	63 (38.0)		
61–70	82 (30.5)	7 (16.3)	16 (26.7)	59 (35.5)		
≥ 71	56 (20.8)	6 (14.0)	6 (10.0)	44 (26.5)		
BMI					1.795^a^	0.773
< 18.5	12 (4.5)	1 (2.3)	2 (3.3)	9 (5.4)		
18.5–25.0	177 (65.8)	31 (72.1)	38 (63.3)	108 (65.1)		
≥ 25.0	80 (29.7)	11 (25.6)	20 (33.3)	49 (29.5)		
Ethnicity					0.362^a^	0.834
Han	253 (94.1)	41 (95.3)	57 (95.0)	155 (93.4)		
Ethnic Minority	16 (5.9)	2 (4.7)	3 (5.0)	11 (6.6)		
Residence Type					16.651^a^	0.002
Urban	129 (48.0)	22 (51.2)	36 (60.0)	71 (42.8)		
Urban-rural	52 (19.3)	12 (27.9)	14 (23.3)	26 (15.7)		
Rural	88 (32.7)	9 (20.9)	10 (16.7)	69 (41.6)		
Marital Status					7.807^a^	0.253
Married	177 (65.8)	32 (74.4)	44 (73.3)	101 (60.8)		
Divorced	31 (11.5)	6 (14.0)	6 (10.0)	19 (11.4)		
Widowed	56 (20.8)	4 (9.3)	9 (15.0)	43 (25.9)		
Unmarried	5 (1.9)	1 (2.3)	1 (1.7)	3 (1.8)		
Living Arrangement					11.168^a^	0.025
With spouse	114 (42.4)	21 (48.8)	28 (46.7)	65 (39.2)		
With children/parents	58 (21.6)	7 (16.3)	5 (8.3)	46 (27.7)		
Other	97 (36.1)	15 (34.9)	27 (45.0)	55 (33.1)		
Education Level					38.904^a^	< 0.001
Primary school	177 (65.8)	16 (37.2)	32 (53.3)	129 (77.7)		
Junior high school	80 (29.7)	20 (46.5)	25 (41.7)	35 (21.1)		
High school and above	12 (4.5)	7 (16.3)	3 (5.0)	2 (1.2)		
Employment Status					48.212^a^	< 0.001
Employed	82 (30.5)	27 (62.8)	34 (48.3)	26 (15.7)		
Retired	84 (31.2)	9 (20.9)	12 (20.0)	63 (38.0)		
Unemployed	103 (38.3)	7 (16.3)	19 (31.7)	77 (46.4)		
Personal Monthly Income					96.369^a^	< 0.001
≤ 1000	169 (62.8)	13 (30.2)	19 (31.7)	137 (82.5)		
1001–3000	55 (20.4)	9 (20.9)	30 (50.0)	16 (9.6)		
≥ 3001	45 (16.7)	21 (48.8)	11 (18.3)	13 (7.8)		
Household Monthly Income per Capita					31.699^a^	< 0.001
≤ 1000	91 (33.8)	8 (18.6)	9 (15.0)	74 (44.6)		
1001–3000	116 (43.1)	16 (37.2)	33 (55.0)	67 (40.4)		
≥ 3001	62 (23.0)	19 (44.2)	18 (30.0)	25 (15.1)		
Number of Children					4.703^a^	0.095
≤ 2	179 (66.5)	33 (76.7)	34 (56.7)	112 (67.5)		
> 2	90 (33.5)	10 (23.3)	26 (43.3)	54 (32.5)		
Exercise Frequency (≥ 30 min/day)					11.342^a^	0.078
Daily	77 (28.6)	16 (37.2)	21 (35.0)	40 (24.1)		
Often	30 (11.2)	6 (14.0)	10 (16.7)	14 (8.4)		
Occasionally	87 (32.3)	14 (32.6)	15 (25.0)	58 (34.9)		
Never	75 (27.9)	7 (16.3)	14 (23.3)	54 (32.5)		
Smoking History					3.519^a^	0.172
No	139 (51.7)	18 (41.9)	28 (46.7)	93 (56.0)		
Yes	130 (48.3)	25 (58.1)	32 (53.3)	73 (44.0)		
Drinking History					7.266^a^	0.026
No	185 (68.8)	23 (53.5)	39 (65.0)	123 (74.1)		
Yes	84 (31.2)	20 (46.5)	21 (35.0)	43 (25.9)		
Health Insurance Type					11.458^a^	0.003
Urban Resident Insurance	242 (90.0)	33 (76.7)	53 (88.3)	156 (94.0)		
Employee Insurance	27 (10.0)	10 (23.3)	7 (11.7)	10 (6.0)		
Complications					8.599^a^	0.072
0 types	164 (61.0)	31 (72.1)	43 (71.7)	90 (54.2)		
1 type	68 (25.3)	8 (18.6)	12 (20.0)	48 (28.9)		
2 or more types	37 (13.8)	4 (9.3)	5 (8.3)	28 (16.9)		
HIV Diagnosis Year					3.628^a^	0.459
< 3 years	102 (37.9)	21 (20.6)	23 (22.5)	58 (56.9)		
3–5 years	114 (42.4)	17 (14.9)	25 (21.9)	72 (63.2)		
> 5 years	53 (19.7)	5 (9.4)	12 (22.6)	36 (67.9)		
Treatment Plan					6.304^a^	0.178
EFV/TDF/3TC	117 (43.5)	25 (58.1)	26 (43.3)	66 (39.8)		
Biktarvy	107 (39.8)	15 (34.9)	22 (36.7)	70 (42.2)		
Other	45 (16.7)	3 (7.0)	12 (20.0)	30 (18.1)		
CD4 + Lymphocyte Count					5.733^a^	0.220
≤ 200	37 (13.8)	8 (18.6)	9 (15.0)	20 (12.0)		
201–500	120 (44.6)	21 (48.8)	20 (33.3)	79 (47.6)		
≥ 500	112 (41.6)	14 (32.6)	31 (51.7)	67 (40.4)		
HIV Viral Load					0.521^a^	0.771
≤ 200	234 (87.0)	36 (83.7)	53 (88.3)	145 (87.3)		
> 200	35 (13.0)	7 (16.3)	7 (11.7)	21 (12.7)		
Frailty	5 (4, 5)	5 (5, 5)	5 (5, 5)	5 (4, 5)	15.285^b^	< 0.001
Social Support	36 (31, 39)	38 (34, 42)	38 (31, 41)	35 (30, 38)	19.739^b^	< 0.001

EFV/TDF/3TC: Efavirenz + Tenofovir + Lamivudine

Class 1 = Low SPA group

Class 2 = Moderate SPA group

Class 3 = High SPA group

^a^Represents the χ² value (Chi-square test)

^b^Represents the H value (Kruskal-Wallis H test)

### Latent class classification of SPA among older PLWH

A series of latent profile models with 1 to 5 classes were fitted to identify the optimal classification of SPA among older PLWH. The model fit indices are presented in Table 3.

**Table 3:** Fit indices for latent profile analysis of SPA subgroups (*n* = 269)

Model	AIC	BIC	aBIC	Entropy	LMR	BLRT	Class Probabilities (%)
1	7731.100	7767.047	7735.341	-	-	-	100
2	7063.610	7121.125	7070.395	0.934	< 0.001	< 0.001	30.112 / 69.888
3	6909.633	6988.717	6918.963	0.920	0.0157	< 0.001	15.985 / 22.305 / 61.710
4	6780.008	6880.660	6791.882	0.962	0.0018	< 0.001	14.870 / 15.613 / 8.178 / 61.338
5	6730.045	6852.265	6744.463	0.937	0.2891	< 0.001	14.498 / 56.877 / 13.011 / 8.178 / 7.435

*AIC* Akaike Information Criterion, *BIC* Bayesian Information Criterion, *aBIC* Adjusted Bayesian Information Criterion, *LMR* Lo-Mendell-Rubin likelihood ratio test, *BLRT* Bootstrapped likelihood ratio test

The results showed that AIC, BIC, and aBIC values decreased progressively as the number of latent classes increased, indicating improved model fit. However, the five-class model was not supported, as the LMR test was not statistically significant (*P* > 0.05), suggesting no significant improvement compared with the four-class model. Overall, the three-class model demonstrated the best balance of model fit and classification quality, with an entropy value of 0.920, and significant LMR and BLRT results (*P* < 0.05), indicating high classification accuracy and good model fit.

In terms of class distribution, the three-class solution showed better stability and interpretability than the four-class model. Specifically, in the four-class model, the smallest class accounted for less than 10% of the sample (*n* = 22), which was smaller than the smallest class in the three-class model (15.985%, *n* = 43). The three classes consisted of 43, 60, and 166 participants, respectively. Although one class contained slightly fewer than 50 participants, model selection also considered its practical relevance in capturing SPA patterns among PLWH.

To validate classification accuracy, discriminant analysis was conducted. The average posterior probabilities for the three-class model ranged from 92.3% to 97.7%, further confirming good classification reliability and discriminant validity (see Table 4). Therefore, the three-class solution was selected as the optimal latent classification model for SPA in older PLWH.

**Table 4:** Probability matrix for the three latent profiles of SPA

Classification of latent profiles	Probability of belonging to profiles (%)		
	Class 1	Class 2	Class 3
Class 1	97.0	3.0	0
Class 2	2.8	92.3	4.9
Class 3	0	2.3	97.7

Class 1 = Low SPA group

Class 2 = Moderate SPA group

Class 3 = High SPA group

### Naming of latent SPA profiles among older PLWH

**Fig. 3:**
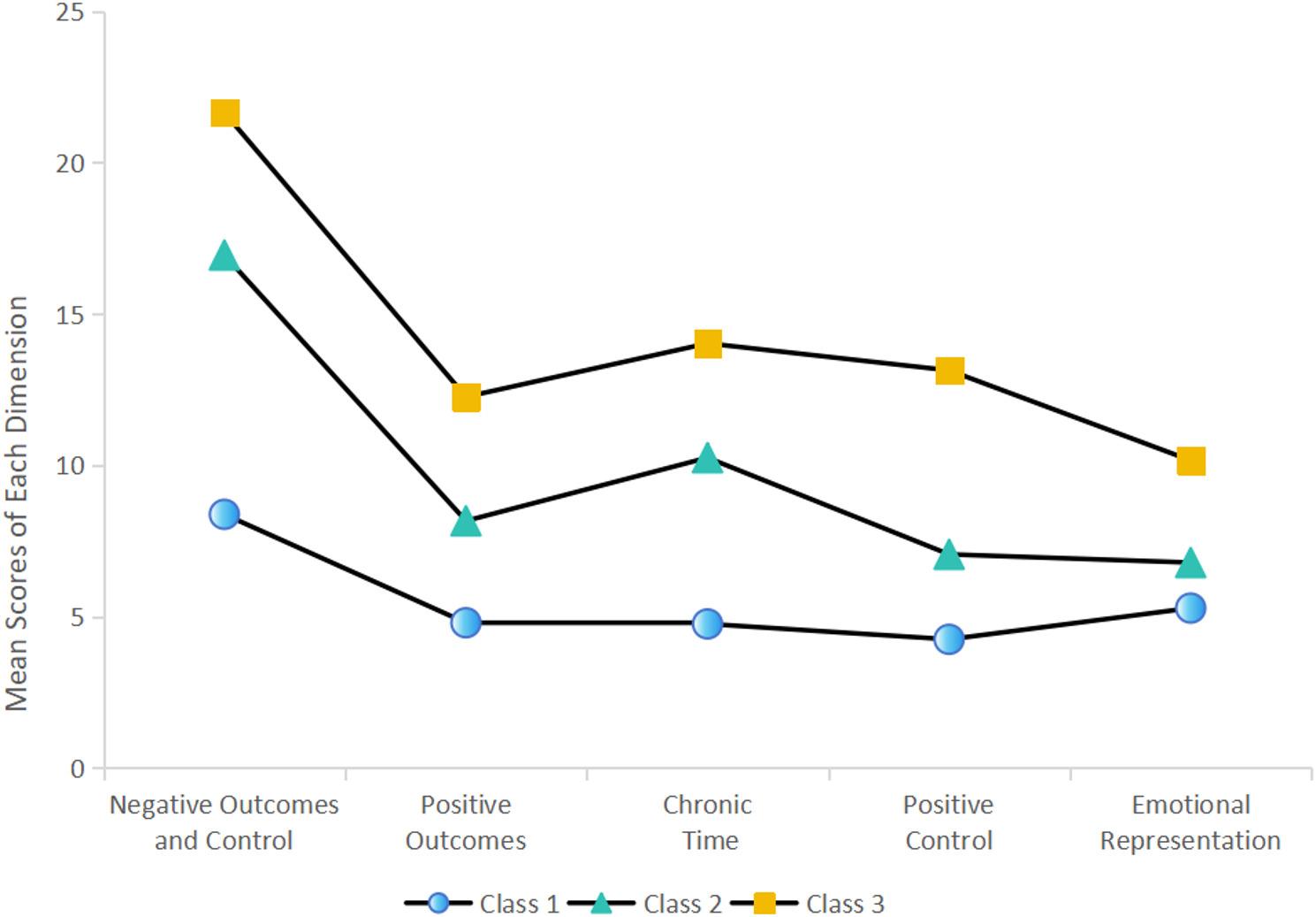
Latent profile model of SPA in Older PLWH patients

As shown in Fig. 3; Table 5, the three latent classes were named according to their score patterns across all B-APQ dimensions. 

**Table 5:** B-APQ total score and dimension scores across latent classes (*N* = 269)

Dimension	Class 1	Class 2	Class 3	H	*P*
Negative consequences and control	8.00 (7.00, 9.50)	17.00(15.00,19.00)	22.00(20.00,24.00)	155.360	< 0.001
Positive consequences	5.00 (4.00, 6.00)	7.50 (6.00, 10.00)	13.00(11.00,14.00)	147.843	< 0.001
Chronic timeline	5.00 (3.00, 6.00)	10.00(7.00, 13.00)	15.00(13.00,15.00)	154.862	< 0.001
Positive control	4.00 (3.00, 5.00)	6.00 (4.00, 9.50)	14.00(12.00,15.00)	160.927	< 0.001
Emotional representation	5.00 (4.00, 6.50)	7.00 (4.00, 9.00)	10.50 (8.00, 13.00)	84.943	< 0.001
Total self-perceptions of ageing score	27.00(24.00,31.50)	49.00(44.00,53.50)	71.00 (66.00, 76.00)	199.658	< 0.001

Class 1 = Low SPA group

Class 2 = Moderate SPA group

Class 3 = High SPA group

Class 1 (*n* = 43, 15.985%) had the lowest scores across all B-APQ dimensions, with a total score of 27.00 (24.00–31.50). Notably, this class showed the lowest score in the positive control dimension (reverse-scored), indicating a more positive perception of ageing, fewer perceived negative effects of ageing, and stronger self-regulation and adaptation capacity. Therefore, this class was labelled the low SPA group.

Class 2 (*n* = 60, 22.305%) demonstrated moderate scores across all dimensions, with a total score of 49.00 (44.00–53.50), reflecting intermediate characteristics of ageing perceptions. This class was therefore labelled the moderate SPA group.

Class 3 (*n* = 166, 61.710%), the largest subgroup, showed higher scores across all B-APQ dimensions, with a total score of 71.00 (66.00–76.00). In particular, the highest scores were observed in the “negative consequences and control” dimension, suggesting more pessimistic and negative attitudes toward ageing and a stronger perception of adverse ageing-related outcomes. This class was therefore labelled the high SPA group.

### Univariate analysis of SPA among older PLWH

χ² tests showed that there were significant differences among the SPA latent classes in age (χ² = 21.948, *P* < 0.001), residence type (χ² = 16.651, *P* = 0.002), living arrangement (χ² = 11.168, *P* = 0.025), educational level (χ² = 38.904, *P* < 0.001), employment status (χ² = 48.212, *P* < 0.001), monthly personal income (χ² = 96.369, *P* < 0.001), Household Monthly Income per Capita (χ² = 31.699, *P* < 0.001), alcohol history (χ² = 7.266, *P* = 0.026), and Urban Resident Insurance (χ² = 11.458, *P* = 0.003). In addition, Kruskal–Wallis H tests indicated significant differences among the three classes in frailty (H = 15.285, *P* < 0.001) and social support (H = 19.739, *P* < 0.001), as shown in Table 2.

### Multicollinearity analysis

Prior to multinomial logistic regression analysis, multicollinearity was assessed for variables showing statistically significant differences in univariate analyses. A tolerance < 0.10 and VIF > 10.0 were used as criteria for multicollinearity [59]. No evidence of multicollinearity was detected among the included variables. All tolerance values were > 0.10 and all VIF values were < 10.0, indicating no significant multicollinearity among predictors. Therefore, all statistically significant variables were included in the subsequent multinomial logistic regression analysis. Results are presented in Table 6.

**Table 6:** Multicollinearity analysis among independent variables

Variables	tolerance	VIF
Age	0.825	1.212
Residence Type	0.895	1.117
Living Arrangement	0.670	1.492
Education Level	0.769	1.300
Employment Status	0.667	1.500
Personal Monthly Income	0.370	2.699
Household Monthly Income per Capita	0.573	1.744
alcohol history	0.953	1.050
Urban Resident Insurance	0.663	1.509
Frailty	0.825	1.212
Social Support	0.538	1.859

### Multinomial logistic regression analysis of factors associated with SPA among older PLWH

In this study, SPA latent class membership was used as the dependent variable. Using the low SPA group (Class 1) as the reference category, variables that showed statistically significant differences in univariate analyses were entered into a multinomial logistic regression model. The results are presented in Table 7.

**Table 7:** Multinomial logistic regression for predicting SPA variables in older PLWH

	Characteristic (Reference Group)	Class 2 VS Class 1				Class 3 VS Class 1			
		β	S.E.	OR (95% CI)	*P*	β	S.E.	OR (95% CI)	*P*
Age	≥ 71								
50–60		0.394	0.893	1.482 (0.257, 8.536)	0.660	-1.231	0.839	0.292 (0.056, 1.514)	0.143
61–70		1.118	0.921	3.057 (0.503, 18.596)	0.225	0.818	0.848	2.265 (0.430, 11.935)	0.335
Residence Type	Rural								
Urban		0.380	0.633	1.463 (0.423, 5.054)	0.548	-0.484	0.582	0.616 (0.197, 1.927)	0.405
Urban-rural		-0.122	0.701	0.885 (0.224, 3.497)	0.862	-0.994	0.656	0.370 (0.102, 1.339)	0.130
Living Arrangement	Other								
With spouse		-0.122	0.701	0.885 (0.224, 3.497)	0.862	0.249	0.593	1.282 (0.401, 4.097)	0.675
With children /parents		-1.253	0.758	0.286 (0.065, 1.262)	0.098	0.214	0.670	1.238 (0.333, 4.601)	0.750
Education Level	High school and above								
Primary school		1.568	0.985	4.796 (0.696, 33.065)	0.111	3.888	1.234	48.798 (4.345, 548.017)	0.002
Junior high school		0.764	0.921	2.146 (0.353, 13.042)	0.407	2.426	1.191	11.313 (1.097, 116.685)	0.042
Employment Status	Unemployed								
Employed		-1.135	0.727	0.321 (0.077, 1.337)	0.119	-1.308	0.654	0.270 (0.075, 0.974)	0.045
Retired		-0.419	0.873	0.658 (0.119, 3.637)	0.631	-0.261	0.812	0.771 (0.157, 3.786)	0.748
Personal Monthly Income	≥ 3001								
≤ 1000		0.877	0.972	2.404 (0.357, 16.168)	0.367	1.872	0.925	6.504 (1.061, 39.853)	0.043
1001–3000		2.126	0.786	8.382 (1.796, 39.128)	0.007	1.021	0.818	2.777 (0.559, 13.794)	0.212
Household Monthly Income per Capita	≥ 3001								
≤ 1000		-1.414	0.967	0.243 (0.037, 1.617)	0.144	-1.091	0.906	0.336 (0.057, 1.984)	0.229
1001–3000		-0.734	0.752	0.480 (0.110, 2.098)	0.329	-0.992	0.772	0.371 (0.082, 1.684)	0.199
Alcohol History	Yes								
No		0.480	0.505	1.615 (0.601, 4.345)	0.342	1.235	0.499	3.438 (1.292, 9.150)	0.013
Health Insurance Type	Employee Insurance								
Urban Resident Insurance		0.426	0.816	1.531 (0.309, 7.586)	0.602	-0.030	0.847	0.971 (0.185, 5.104)	0.972
Social Support Score		-0.002	0.046	0.998 (0.911, 1.093)	0.962	-0.082	0.048	0.922 (0.839, 1.013)	0.089
Frailty Score		-0.947	0.423	0.388 (0.169, 0.888)	0.025	-0.885	0.433	0.413 (0.177, 0.965)	0.041

*OR*  Odds Ratio

Class 1 = Low SPA group

Class 2 = Moderate SPA group

Class 3 = High SPA group

Educational level was significantly associated with SPA classification among older PLWH. Compared with participants with a high school education or above, those with junior high school education (Class 3 vs. Class 1: OR = 48.798, *P* = 0.002) and primary school education or below (Class 3 vs. Class 1: OR = 11.313, *P* = 0.042) were more likely to be classified into the high SPA group.Employment status was also significantly associated with SPA profiles. Compared with unemployed participants, those who were employed had a significantly lower likelihood of being classified into the high SPA group (Class 3 vs. Class 1: OR = 0.270, *P* = 0.045).Alcohol consumption was associated with SPA classification. Participants without a history of alcohol consumption were more likely to be classified into the high SPA group compared with those with alcohol consumption (Class 3 vs. Class 1: OR = 3.438, *P* = 0.013).Monthly personal income showed a significant association with SPA classification. Compared with participants with a monthly income ≥ 3001 RMB, those with a monthly income ≤ 1000 RMB were more likely to be classified into the high SPA group (Class 3 vs. Class 1: OR = 6.504, *P* = 0.043), whereas those with a monthly income of 1001–3000 RMB were more likely to be classified into the moderate SPA group (Class 2 vs. Class 1: OR = 8.382, *P* = 0.007).

In addition, frailty was negatively associated with SPA classification. Each one-unit increase in frailty score was associated with a lower likelihood of being classified into either the moderate SPA group (Class 2 vs. Class 1: OR = 0.388, *P* = 0.025) or the high SPA group (Class 3 vs. Class 1: OR = 0.413, *P* = 0.041).

### Association between SPA and interoception in older PLWH

The median interoception scores in the “low self-perceptions of ageing group”, “moderate self-perceptions of ageing group”, and “high self-perceptions of ageing group” were 34.00 (19.56–36.25), 20.27 (17.40–34.10), and 11.81 (8.57–19.25), respectively. The Kruskal–Wallis H test indicated statistically significant differences in total interoception scores and all subscale scores across the three groups (*P* < 0.001 ), see Table 8.

**Table 8:** Differences in interoception scores between different self-perception of aging classes

Dimension	Class 1	Class 2	Class 3	H	*P*
Noticing	4.50 (2.50, 4.75)	2.88 (1.50, 4.50)	1.25 (0.50, 2.25)	67.912	< 0.001
Not-Distracting	3.67 (2.33, 4.33)	2.59 (2.00, 4.00)	1.33 (0.83, 2.17)	63.735	< 0.001
Not-Worrying	4.00 (2.80, 4.40)	2.60 (1.85, 4.00)	1.40 (0.95, 2.40)	63.687	< 0.001
Attention Regulation	4.00 (2.43, 4.71)	2.71 (2.14, 4.43)	1.57 (1.29, 2.57)	63.313	< 0.001
Emotional Awareness	4.00 (2.60, 4.40)	2.80 (2.40, 4.20)	1.40 (1.00, 2.40)	65.372	< 0.001
Self-Regulation	4.00 (2.50, 4.50)	2.75 (2.25, 4.50)	1.50 (1.25, 2.75)	56.904	< 0.001
Body Listening	4.33 (2.33, 4.67)	2.67 (2.00, 4.33)	1.67 (1.33, 2.67)	57.275	< 0.001
Trusting	4.33 (2.33, 4.67)	2.84 (2.00, 4.33)	1.67 (1.00, 2.67)	60.389	< 0.001
Total Interoceptive Score	34.00 (19.56, 36.25)	20.27 (17.40, 34.10)	11.81 (8.57, 19.25)	69.067	< 0.001

Class 1 = Low SPA group

Class 2 = Moderate SPA group

Class 3 = High SPA group

To control for potential confounding effects of sociodemographic and disease-related variables, multivariable quantile regression analyses were further conducted. Compared with the low SPA group, participants in the high SPA group had significantly lower total interoception scores and lower scores across all dimensions. The adjusted median difference in total interoception score was − 38.00 (95% CI: −52.56 to − 23.44; *P* < 0.001). In contrast, no statistically significant differences were observed between the moderate and low SPA groups, with an adjusted median difference of − 5.91 (95% CI: −20.29 to 8.47; *P* = 0.419), see Table 9.

**Table 9:** Multivariable quantile regression analysis of total and domain-specific interoception scores across SPA classes

Dimension	Reference group (Class 1)	Median difference	95% CI	*P*
Noticing				
	Class 2	-0.066	(-0.550, 0.417)	0.787
	Class 3	-1.265	(-1.754, -0.775)	< 0.001
Not-Distracting				
	Class 2	0.072	(-0.437, 0.582)	0.780
	Class 3	-0.964	(-1.480, -0.448)	< 0.001
Not-Worrying				
	Class 2	-0.472	(-0.944, 0.001)	0.050
	Class 3	-1.436	(-1.915, -0.958)	< 0.001
Attention Regulation				
	Class 2	-0.134	(-0.557, 0.289)	0.534
	Class 3	-0.751	(-1.179, -0.323)	0.001
Emotional Awareness				
	Class 2	-0.143	(-0.625, 0.339)	0.560
	Class 3	-0.989	(-1.477, -0.502)	< 0.001
Self-Regulation				
	Class 2	-0.002	(-0.478, 0.474)	0.993
	Class 3	-0.857	(-1.339, -0.375)	0.001
Body Listening				
	Class 2	-0.211	(-0.660, 0.238)	0.356
	Class 3	-0.710	(-1.165, -0.255)	0.002
Trusting				
	Class 2	0.048	(-0.529, 0.434)	0.845
	Class 3	-0.909	(-1.397, -0.422)	< 0.001
Total Interoceptive Score				
	Class 2	-5.912	(-20.294, 8.469)	0.419
	Class 3	-38.000	(-52.558, -23.442)	< 0.001

The model was adjusted for age, place of residence, living arrangement, educational level, employment status, personal monthly income, household per capita monthly income, alcohol consumption history, type of medical insurance, frailty score, and social support score

Class 1 = Low SPA group

Class 2 = Moderate SPA group

Class 3 = High SPA group

**Fig. 4:**
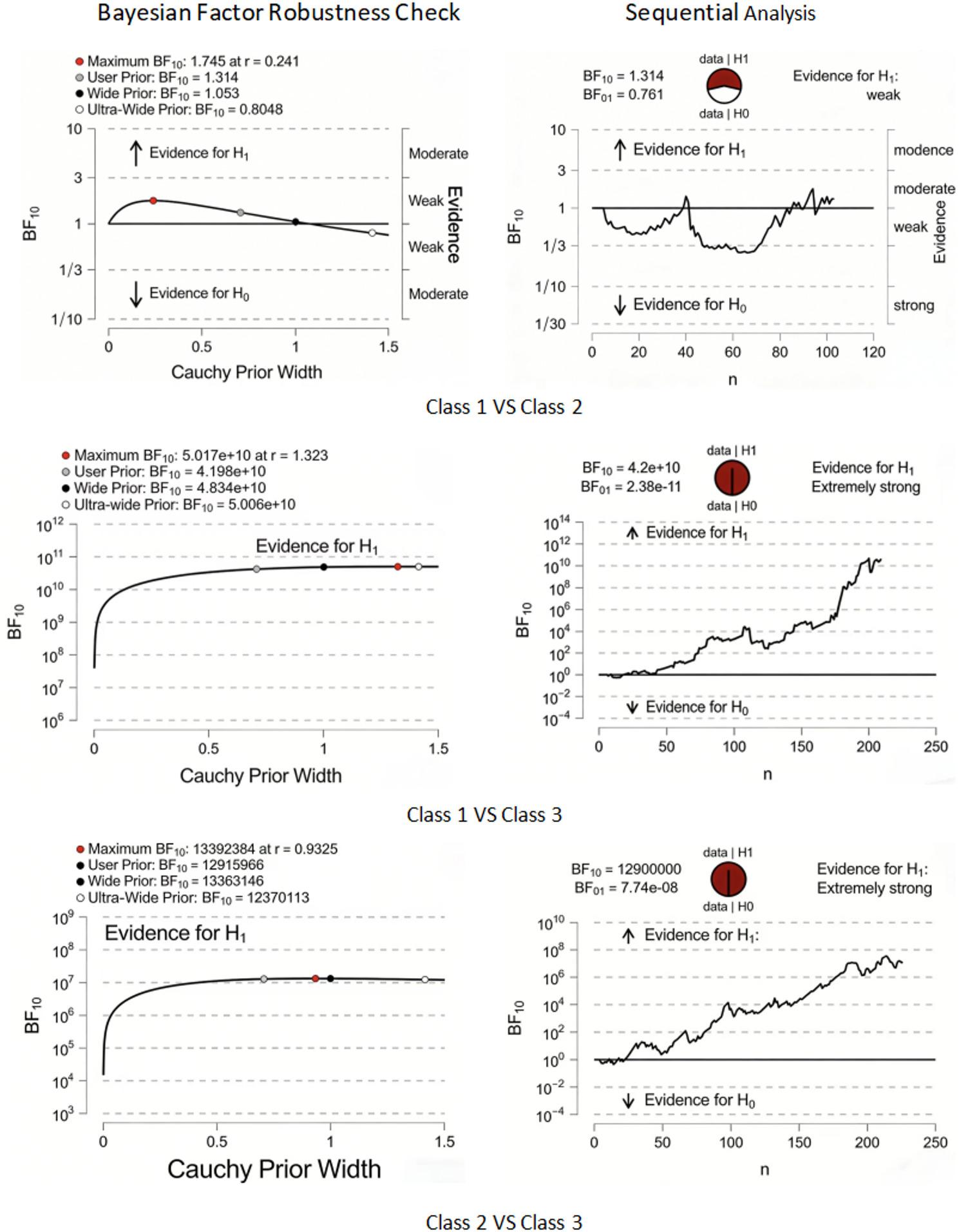
Bayesian factor robustness check and sequential analysis based on three different profiles

**Fig. 5:**
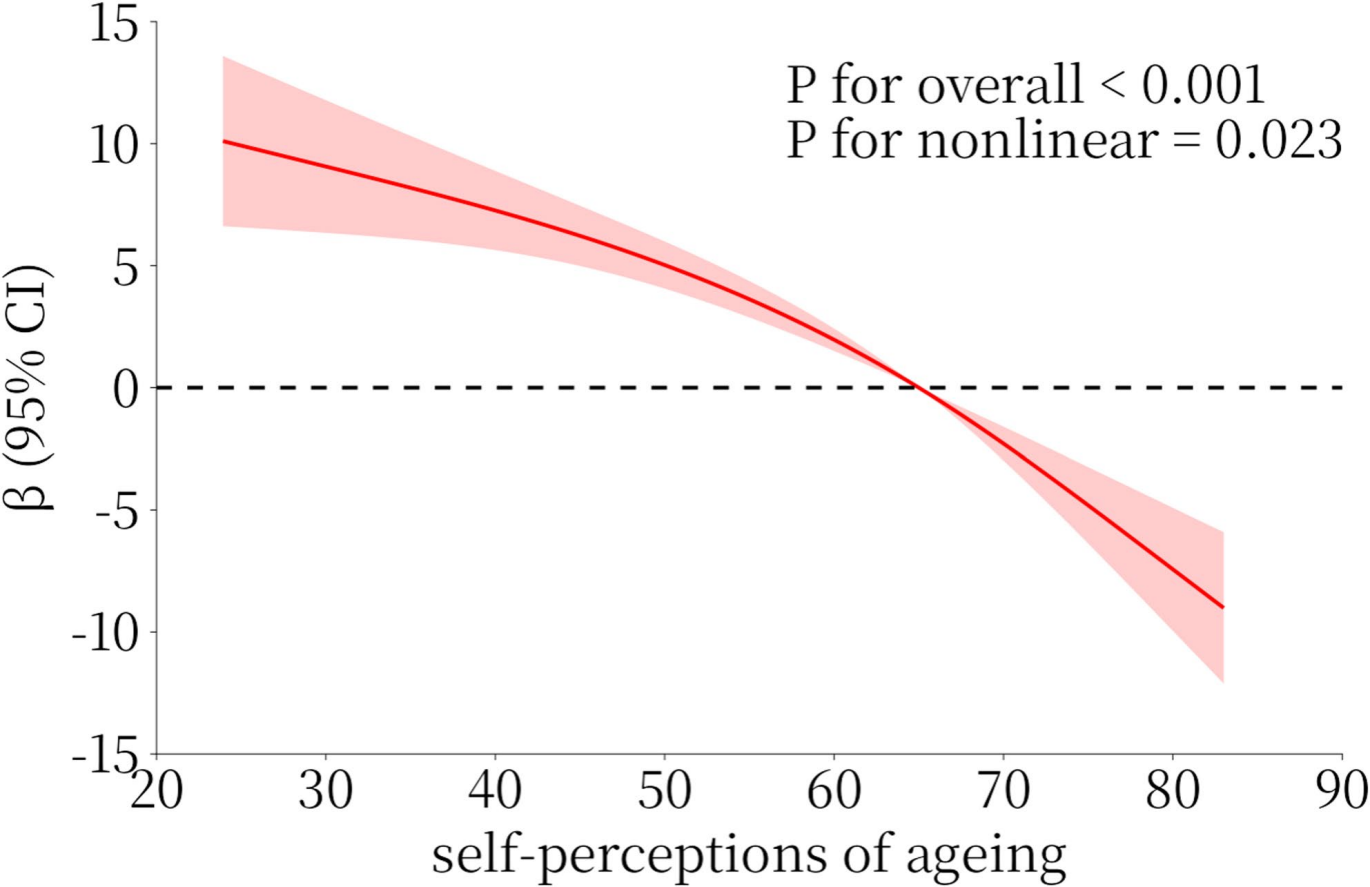
RCS analysis of the non-linear relationship between SPA and interoception in older PLWH

To further assess the robustness of these findings, Bayesian factor analyses were performed. Strong evidence supported differences between the low and high SPA groups (BF10 = 4.2 × 10¹⁰) and between the moderate and high groups (BF10 = 12,900,000), indicating substantially lower interoception in the high SPA group. In contrast, evidence for differences between the low and moderate groups was weak (BF10 = 1.305), suggesting insufficient support for a true difference between these two groups (see Fig. 4). RCS regression further demonstrated a significant nonlinear association between SPA and interoception (P for nonlinearity = 0.023), with a statistically significant overall model fit (*P* < 0.05), see Fig. 5. Threshold analysis identified a critical inflection point at a B-APQ score of 25.55, above which interoception declined more sharply. Detailed results are presented in Table 10.

**Table 10:** Threshold effect analysis of the relationship between SPA and interoception in older PLWH

Outcome	effect	*P*
Model 1 Fitting model by standard linear regression	-0.31 (-0.38 - -0.25)	< 0.001
Model 2 Fitting model by two-piecewise linear regression		
Inflection point	25.55	
<25.55	0.96 (-0.02–1.95)	0.078
≥25.55	-0.37 (-0.44 - -0.30)	< 0.001
P for likelihood test		< 0.001

## Discussion

### Heterogeneity of SPA among older PLWH

In this study, latent profile analysis identified three distinct subgroups of SPA among older PLWH, namely the low SPA group (Class 1), moderate SPA group (Class 2), and high SPA group (Class 3), accounting for 15.985%, 22.305%, and 61.710% of the sample, respectively. These findings indicate substantial heterogeneity in ageing perceptions among older PLWH, suggesting that traditional variable-centered approaches based on total scale scores may be insufficient to capture such underlying differences.

The high SPA group accounted for the largest proportion of participants (61.710%) and exhibited consistently higher scores across all B-APQ dimensions, particularly in “negative consequences and control” and “chronic timeline”. This pattern suggests that individuals in this group are more likely to perceive ageing as a persistent and irreversible process and to associate it with functional decline, reduced social roles, and adverse health outcomes. Moreover, they tend to report lower perceived control over age-related changes. Previous studies have shown that persistent negative ageing beliefs may influence health outcomes through psychological, behavioral, and physiological pathways, contributing to depression, functional decline, and reduced quality of life [60–62]. For older PLWH, such negative cognitions may be further reinforced by HIV-related accelerated ageing and long-term disease-related stigma. Therefore, healthcare providers should prioritize this subgroup in clinical practice and implement targeted interventions such as cognitive restructuring and enhancement of self-efficacy to improve ageing perceptions.

The low SPA group demonstrated a generally positive pattern of ageing perceptions, characterized by lower perceived negative consequences and stronger confidence in managing age-related changes. Notably, this group showed the lowest scores in the “positive control” dimension, suggesting a greater tendency to perceive themselves as capable of actively managing physical and life changes associated with ageing. This finding is consistent with previous evidence indicating that positive SPA serves as a protective factor for both physical and mental health in older adults, contributing to higher life satisfaction, better physical functioning, and reduced psychological distress [13, 63]. Therefore, for individuals in this subgroup, efforts should focus on maintaining their positive ageing attitudes and promoting successful ageing trajectories.

The moderate SPA group exhibited intermediate scores across all dimensions, indicating a transitional cognitive state without clearly positive or negative ageing perceptions. Such a profile may reflect a more fluid and potentially modifiable stage of ageing cognition, making this group particularly sensitive to environmental influences and interventions. Previous studies relying on mean or median-based classification methods have often failed to identify this subgroup, thereby overlooking its unique psychological characteristics and intervention needs [32, 64]. Based on the present findings, individuals in the moderate SPA group may represent a key target population for intervention. Future longitudinal studies are warranted to explore whether this subgroup may transition toward more positive or negative profiles in response to disease progression, changes in social support, or psychological interventions, thereby informing the development of stratified and dynamic intervention strategies.

### Factors associated with SPA among older PLWH based on the HEM

Based on the HEM, this study examined factors associated with SPA among older PLWH from multiple dimensions, including individual characteristics, behavioural factors, and living and working conditions.

### Individual characteristics

At the individual level, frailty was identified as a significant factor associated with SPA among older PLWH. Previous studies have generally considered frailty a risk factor for negative SPA [65]. However, in the present study, higher frailty scores were unexpectedly associated with a greater likelihood of being classified into the low SPA group. This finding differs from traditional evidence and suggests that unique cognitive mechanisms may exist in older PLWH. One possible explanation is that PLWH may demonstrate distinct patterns of health attribution. Prior research has shown that PLWH often exhibit an age-related cognitive bias, tending to perceive themselves as not yet entering old age [66]. Consequently, even when experiencing typical frailty manifestations such as reduced muscle mass and declining physical capacity, they may attribute these symptoms to HIV infection or long-term antiretroviral therapy rather than to normal ageing processes [67]. Such attributional bias may weaken the association between frailty and negative ageing perceptions. This finding highlights the importance for healthcare professionals to identify high-risk individuals through frailty screening and to help patients distinguish between disease-related symptoms and normal ageing processes, thereby fostering more positive ageing perceptions.

### Behavioural characteristics

At the behavioural level, participants with a history of alcohol consumption were less likely to report negative SPA. Previous studies suggest that alcohol use, in certain sociocultural contexts, is often associated with social activities that facilitate interpersonal interaction and social participation. Social support has been widely recognized as a protective factor for SPA [68]. In addition, moderate socially embedded drinking may alleviate loneliness and psychological stress, thereby indirectly influencing subjective perceptions of ageing [69]. However, alcohol consumption should not be interpreted as a recommended intervention for promoting positive ageing. The observed association is more likely to reflect the social interaction context linked to drinking behaviour rather than a protective effect of alcohol itself. Therefore, clinical practice should focus on enhancing social participation and strengthening social support rather than encouraging alcohol consumption.

### Living and working conditions

At the level of socioeconomic and living conditions, lower educational attainment, unemployment, and lower personal monthly income were associated with a higher likelihood of being classified into the moderate or high SPA groups among older PLWH. Individuals with higher education levels generally possess better health literacy and access to health-related information, which may contribute to more positive ageing perceptions [15, 70]. In this study, the relatively high proportion of participants with primary education or below may be related to the older age structure of the sample. These findings suggest that healthcare providers should adopt simple and accessible health education strategies for individuals with lower educational attainment to improve understanding of disease management and ageing-related knowledge, thereby reducing uncertainty and negative perceptions. In 2025, the Chinese government issued the Opinions of the CPC Central Committee and the State Council on Deepening the Reform and Development of Elderly Care Services, which emphasized establishing an integrated elderly education system and promoting blended online–offline education through community and elderly learning institutions. This policy provides important structural support for improving ageing perceptions among older PLWH. Employment status was also a significant determinant of SPA. Compared with unemployed individuals, those who were employed were less likely to be classified into the high SPA group. Employment provides not only financial stability but also social roles, a sense of value, and self-identity, which may alleviate psychological distress associated with ageing and chronic illness [71]. In contrast, unemployment may weaken social roles and reduce available resources, thereby reinforcing negative ageing perceptions [72]. Furthermore, lower income was significantly associated with more negative SPA, consistent with previous studies [73]. Individuals with lower income often experience greater financial pressure related to healthcare costs and daily living, which may intensify concerns about health deterioration and ageing.Although China has implemented the “Four Frees and One Care” policy, which provides free antiretroviral therapy, financial assistance, and support for livelihood development among PLWH, some older individuals may still experience persistent economic vulnerability.

Overall, older PLWH with lower educational attainment, unemployment, and low income represent a high-risk population for negative SPA. Targeted interventions, including health education, psychological support, and integration of social resources, should be prioritized to improve ageing perceptions and promote positive self-management behaviours, ultimately enhancing quality of life.

### Association between SPA and interoception in older PLWH

This study found significant differences in interoception levels across different SPA latent classes among older PLWH, with a general trend indicating that more negative SPA were associated with lower interoception levels. This finding suggests a close relationship between ageing perceptions and the ability to perceive internal bodily signals. A possible explanation is that long-term HIV infection and antiretroviral therapy-related adverse effects may jointly accelerate physiological decline, thereby contributing to more negative ageing perceptions [74]. According to SET [9], internalised negative ageing stereotypes may influence how individuals interpret bodily changes, leading them to ignore, misinterpret, or negatively appraise internal bodily signals. This may subsequently reduce awareness of interoceptive signals such as respiration, heart rate, and somatic sensations. This finding further supports a potential psychophysiological interaction between ageing cognition and bodily self-awareness.

Further RCS regression analysis revealed a significant nonlinear association between SPA and interoception, suggesting that their relationship is not simply linear. Notably, when the B-APQ score reached 25.55, interoception levels began to decline more markedly with increasing SPA, indicating a potential threshold effect. Below this threshold (B-APQ < 25.55), a positive but non-significant association between SPA and interoception was observed. Above this threshold, the direction of the association shifted and became clearly negative. These findings suggest that 25.55 may represent a potential cut-off value for identifying individuals at risk of interoceptive impairment; however, this threshold requires further validation in longitudinal studies and larger samples. Reduced interoception may have important clinical implications. Previous studies have shown that lower interoceptive ability is closely associated with emotional regulation difficulties, as individuals may struggle to accurately identify their emotional states, thereby increasing the risk of anxiety and depression [75]. In addition, impaired interoception may reduce sensitivity to bodily symptoms and treatment-related changes, potentially affecting medication adherence and self-management behaviours [76]. Therefore, for older PLWH with higher SPA levels, comprehensive assessments of interoceptive awareness and psychological status should be considered in clinical practice, along with potential interventions such as mindfulness-based training and body awareness training to improve interoceptive function.

Moreover, Bayesian factor analysis was conducted to assess the robustness of intergroup differences in interoception across SPA classes. The results showed extreme evidence supporting differences between the low and high SPA groups, as well as between the moderate and high SPA groups, indicating that reduced interoception in the high SPA group is a robust and stable finding. In contrast, only weak evidence was observed for differences between the low and moderate SPA groups, suggesting insufficient evidence to confirm a stable distinction between these two groups. This finding implies that the moderate SPA group may represent a transitional stage between positive and negative ageing perceptions. Although their interoceptive levels have not yet significantly declined, their ageing cognition may be more malleable and susceptible to change. Under disease progression or increased psychosocial stress, this group may potentially transition toward the high SPA profile. Therefore, the moderate SPA group may represent a key target population for early psychological intervention and health promotion strategies.

### Limitations

This study has several limitations that should be acknowledged when interpreting the findings. First, the participants were recruited from a single tertiary hospital in a specific region of China using a convenience sampling strategy. This single-center design may introduce selection bias and limit the representativeness of the sample, thereby reducing the generalizability of the findings to PLWH in other regions or cultural contexts. Second, the cross-sectional nature of this study precludes any inference of causal relationships between SPA and interoceptive awareness, and does not allow for the examination of their dynamic changes over time. Longitudinal studies are therefore needed to clarify the temporal and potentially bidirectional relationships between these constructs. Third, all data were collected through self-reported questionnaires, which may be subject to recall bias and social desirability bias, potentially affecting the accuracy of the results. Fourth, although the MAIA-2 demonstrated good internal consistency in this study, its psychometric properties in PLWH have not been fully established. Further validation is required to confirm its reliability, validity, and measurement invariance in this specific population. Finally, although this study incorporated a relatively comprehensive set of variables covering sociodemographic characteristics, clinical factors, and lifestyle behaviors, some potential confounders may not have been fully captured. In particular, prior mental health status and experiences of HIV-related stigma were not assessed, which may also influence SPA and interoceptive processes.

## Conclusion

Based on the HEM, this study is the first to apply LPA to identify three distinct subtypes of SPA among older PLWH, namely the low SPA, moderate SPA, and high SPA groups. These findings indicate substantial heterogeneity in ageing-related cognitions within this population. Educational attainment, employment status, personal monthly income, alcohol history, and frailty were identified as significant factors associated with SPA classification. Furthermore, a significant nonlinear negative association was observed between SPA and interoceptive awareness in older PLWH. A potential threshold effect was identified at a B-APQ score of 25.55, beyond which interoceptive ability declined more sharply with increasing SPA levels. Bayesian factor analyses further confirmed the robustness of the observed differences in interoceptive awareness across SPA subgroups. Collectively, these findings suggest that SPA may not only reflect subjective perceptions of ageing but may also be closely related to individuals’ ability to perceive and interpret internal bodily signals. Stratified health management and individualized psychological interventions tailored to distinct SPA profiles may help improve ageing perceptions and health outcomes in this population. Future multicenter longitudinal studies are warranted to further validate the temporal relationships between SPA and interoceptive awareness, elucidate underlying mechanisms, and evaluate the effectiveness of targeted interventions.

## Data Availability

The data analyzed in this study are not publicly available but available from the corresponding author upon reasonable request.

## Abbreviations

PLWH: People Living with HIV
SPA: Self-Perception of Aging
SET: Stereotype Embodiment Theory
LPA: Latent Profile Analysis
HEM: Health Ecology Model
B-APQ: The Brief Ageing Perceptions Questionnaire
MAIA-2: Multidimensional Assessment of Interoceptive Awareness, Version 2
SSRS: Social Support Rating Scale
